# Effects of time-restricted eating on body composition and metabolic parameters in overweight and obese women: a systematic review and meta-analysis

**DOI:** 10.3389/fnut.2025.1664412

**Published:** 2025-09-16

**Authors:** Shiying Chen, Xiaotian Zhang, Jakub Kortas, Haitao Liu

**Affiliations:** ^1^Department of Sport, Gdansk University of Physical Education and Sport, Gdańsk, Poland; ^2^College of Physical Education, Henan University, Kaifeng, Henan, China

**Keywords:** intermittent fasting, time restricted eating, obesity, metabolism, female, meta-analysis

## Abstract

**Background:**

Time-restricted eating (TRE), a subtype of intermittent fasting, is increasingly explored as a dietary strategy for weight and metabolic management. However, its effects in overweight and obese women remain unclear. This systematic review and meta-analysis aimed to evaluate the impact of TRE on body composition and metabolic parameters in this population.

**Methods:**

Following PRISMA guidelines and PROSPERO registration (CRD42024595472), randomized controlled trials were retrieved from eight English and Chinese databases up to August 2024. Eligible studies included adult women (≥85% female, BMI ≥ 25 kg/m^2^) receiving TRE interventions with or without caloric restriction. Quality was assessed using RoB 2.0 and the PEDro scale. Meta-analyses were conducted using Stata 17.0, with evidence certainty graded using GRADE.

**Results:**

Thirteen RCTs involving 612 participants were included. TRE significantly reduced body weight (WMD = −1.927 kg, 95% CI: −3.688 to −0.166, *p* = 0.032) and fasting insulin levels (WMD = −2.120 μU/mL, 95% CI: −4.172 to −0.069, *p* = 0.043), but showed no significant effects on BMI, fat mass, fat-free mass, visceral fat, blood lipids, glucose, HOMA-IR, or blood pressure (*p* > 0.05). Subgroup analysis revealed greater weight reduction with TRE compared to conventional diets (*p* = 0.046), but not versus calorie restriction alone (*p* = 0.295). No lean mass loss was observed. Four studies reported minor adverse events (e.g., hunger, headache), all self-resolving.

**Conclusion:**

TRE is effective in reducing body weight and lowering fasting insulin in overweight and obese women, without negatively affecting lean body mass. Compared with traditional calorie-restricted diets, TRE does not yield superior weight loss, suggesting comparable efficacy. TRE demonstrates a favorable safety profile and better adherence, supporting its clinical feasibility. Further trials with larger samples and longer follow-up are needed to clarify TRE’s long-term metabolic effects.

**Systematic review registration:**

The systematic review was registered in PROSPERO. Registration ID: CRD42024595472 URL: https://www.crd.york.ac.uk/prospero/display_record.php?RecordID=459547.

## Introduction

The increasing severity of global health issues has positioned obesity and its related chronic diseases as a major public health challenge (1). Obesity not only elevates individual healthcare burdens but also exerts profound socioeconomic impacts. To address this challenge, researchers and healthcare practitioners have persistently sought effective weight management strategies. Traditionally recommended Calorie Restriction (CR) refers to reducing energy intake below the caloric requirement for maintaining current body weight (2). This intervention induces secondary physiological adaptations such as increased appetite, decreased physical activity, and hormonal fluctuations that promote fat deposition while accelerating muscle catabolism (3). Subjects often struggle to maintain long-term adherence to this strategy (4), with prolonged implementation carrying a high risk of weight regain. Observations indicate that adherence to sustained CR begins to decline approximately 1 month post-intervention, with progressive deterioration thereafter (5). Therefore, there is a critical need to develop innovative dietary strategies to manage weight effectively. Beyond energy balance, obesity features chronic low-grade inflammation and metabolic endotoxemia that together impair insulin sensitivity and cardiometabolic health (6, 7). In line with their circadian regulation, meal timing is biologically meaningful (8), motivating evaluation of time-focused dietary strategies such as Intermittent fasting (IF) and TRE.

IF, a dietary regimen comprising regulated fasting-feeding cycles (9), has gained significant scientific attention as a promising weight-management approach (10). Unlike traditional calorie restriction, IF primarily focuses on the temporal regulation of food intake rather than caloric quantity itself (11), including TRE, alternate-day fasting (ADF), and the 5:2 regimen (12). TRE confines eating to a consistent daily window—typically 4–12 h, with the 16:8 pattern being most common—with an extended overnight fast and usually without prescribed caloric restriction, emphasizing when food is eaten rather than how much (13). By contrast, ADF and the 5:2 regimen manipulate intake across days (14). Based on circadian alignment of feeding windows, TRE is classified into two subtypes: early TRE (eTRE) includes breakfast, while delayed TRE (dTRE) shifts the window toward the evening meal.

TRE has been associated with good adherence in free-living conditions, due to its simplicity and minimal need for calorie tracking (15, 16). IF may confer benefits through multiple pathways, including attenuated oxidative stress, improved circadian rhythm regulation, and enhanced ketogenesis (17). Clinical studies in adults indicate that TRE may promote weight loss primarily by reducing energy intake rather than altering energy expenditure, even in the absence of deliberate CR (18). However, evidence regarding the efficacy of TRE remains inconclusive, particularly among women with overweight or obesity. Published meta-analyses have extensively investigated diverse IF regimens, yet most lump TRE with other IF modalities, failing to isolate its specific effects. This conflation likely masks TRE-specific outcomes, impeding a clear understanding of its distinct potential in weight control and metabolic improvement. Over the past three decades, the global prevalence of overweight and obesity in women has risen sharply, with recent estimates indicating that more than half of adult women are affected (19, 20). Coupled with well-documented sex-based differences in metabolic regulation, hormonal responses, and body fat distribution (21), this underscores the need for sex-specific evaluations of TRE in female populations. Moreover, given the documented sex-specific responses (22), dedicated studies are warranted to evaluate TRE’s gender-dimorphic effects. Therefore, this systematic review aims to assess TRE’s effects on anthropometric parameters and metabolic profiles in women (aged ≥ 18 years) with overweight/obesity, with the aim of informing clinical dietary practice and identifying priorities for future research.

## Methods

### Registration and protocol

This systematic review and meta-analysis adhered to the PRISMA (Preferred Reporting Items for Systematic Reviews and Meta-Analyses) guidelines (23), and its protocol is registered on PROSPERO under the number CRD42024595472 (Appendix 1).

### Data source and search strategy

We systematically searched eight electronic databases (PubMed, Embase, Web of Science, Cochrane Library, CNKI, WanFang, VIP, and CBM) from inception through August 11, 2024. The search strategy aimed to identify all published RCTs evaluating the efficacy of TRE (≤12-h eating windows) for improving body composition (primary outcome) and metabolic parameters (secondary outcomes) in adult women (≥18 years) with overweight and obesity (BMI ≥ 25 kg/m^2^). Search terms were systematically developed using the PICO framework: (1) population (e.g., “obesity” “overweight”), (2) intervention (e.g., “time-restricted eating” “intermittent fasting” “TRE”), (3) outcomes (e.g., “weight loss” “body fat”), and (4) study design (e.g., “randomized controlled trial,” “RCT”). We employed a combination of controlled vocabulary (MeSH/Emtree terms) and free-text terms across title/abstract/keyword fields, with language restrictions limited to English and Chinese. The complete search strategy for database is provided in Supplementary Table S1. To ensure comprehensive coverage, the reference lists of identified publications were manually screened to locate studies eligible for inclusion (24).

### Inclusion and exclusion criteria

Inclusion and exclusion criteria are shown in Table 1. Only language (English and Chinese) were applied as restrictions to the search.

**Table 1:** Eligibility criteria based on PICO framework.

	Inclusion criteria	Exclusion criteria
Types of study	•Randomized controlled trials (RCTs, parallel design) •Chinese/English publications	•Reviews/meta-analyses •Animal studies •Conference abstracts without full data
Participants	•Adults >18 years •Female proportion ≥85% •Overweight and obese (BMI ≥ 25)	•Severe comorbidities (cancer, hyperthyroidism, diabetes) •Baseline imbalance (P < 0.05)
Intervention	•Time Restricted Eating	•TRE not verifiable
Comparators	•No intervention •Regular low-calorie diet	•Inter-method comparisons
Outcomes	•Primary: ① Weight change •Secondary: ② BMI ③ FBG ④ TG ⑤ FM ⑥ FFM ⑦ LM ⑧ VFM ⑨ LDL ⑩ HDL ⑪ TC ⑫ FPI ⑬ HOMA-IR ⑭ SBP ⑮ DBP	•Outcomes not reported or qualitatively analyzed only

Intervention (I): TRE was operationalized as a within-day feeding schedule with a pre-specified; Timing (T): no a priori minimum duration; included RCTs ranged 6–52 weeks (see Table 2); BMI, Body Mass Index; FBG, Fasting Blood Glucose; TG, Triglycerides; FM, Fat Mass; FFM, Fat-Free Mass; LM, Lean Mass; VFM, Visceral Fat Mass; LDL, Low-Density Lipoprotein; HDL, High-Density Lipoprotein; TC, Total Cholesterol; FPI, Fasting Plasma Insulin; HOMA-IR, Homeostatic Model Assessment for Insulin Resistance; SBP, Systolic Blood Pressure; DBP, Diastolic Blood Pressure.

### Quality assessment

Two reviewers (ZXT and CSY) evaluated the RoB using Version 2 of the Cochrane risk-of-bias tool (RoB 2) (25). The assessment covered domains including random sequence generation, allocation concealment, blinding of participants, providers and outcome assessment, incomplete outcome data, and selective outcome reporting. Adherence issues were considered under RoB 2 (deviations from intended interventions). Every domain was determined to be of high, moderate, or low RoB. Disagreements were resolved by consensus between CSY and ZXT; if consensus could not be reached, a third reviewer (LHT) adjudicated the final judgment.

The Grading of Recommendations, Assessment, Development and Evaluation approach (GRADE) was employed to assess the certainty of the evidence (26). If sufficient publications were identified (*n* ≥ 10), the funnel-plot (visually asymmetry or not) with Egger’s regression test (*p* < 0.05 indicates the presence of publication bias) was conducted to test publication bias (27).

### Data extraction

The following details were extracted: study characteristics consisting of first author, country, year of publication, study design; demographic characteristics of participants: gender, age, sample size; study design including diet plan, intervention mode and period, additional interventions; outcomes such as weight change, BMI and assessment method; comparators; results of pre–post data, means and standard deviations of targeted outcomes.

### Data synthesis

We separately performed pairwise meta-analyses for each of the primary outcome measures using Stata 17. Weighted mean difference (WMD) was used to establish the effect sizes of TRE compared to the usual care control. Cochrane Q-test and Higgins and Green’s *I^2^* test were used to test heterogeneity. A *p*-value less than 0.05 for Cochrane Q-test or an *I^2^* statistic higher than 50% would indicate the presence of significant heterogeneity. Random-effect analysis would be applied when substantial heterogeneity existed among included studies (28).

### Data presentation

Study characteristics and outcome data were systematically summarized in tables. The primary effect measure was weighted mean difference (WMD) with 95% confidence intervals (CI) for continuous outcomes.

## Results

### Study selection

Our systematic search identified 4,482 records. After removing 1,410 duplicates, we screened 3,072 titles/abstracts, excluding 2,928 irrelevant studies. Full-text review of 144 articles excluded 131 for: ineligible interventions (*n* = 10), wrong settings (*n* = 58), and unavailable outcome data (*n* = 63).

**Figure 1:**
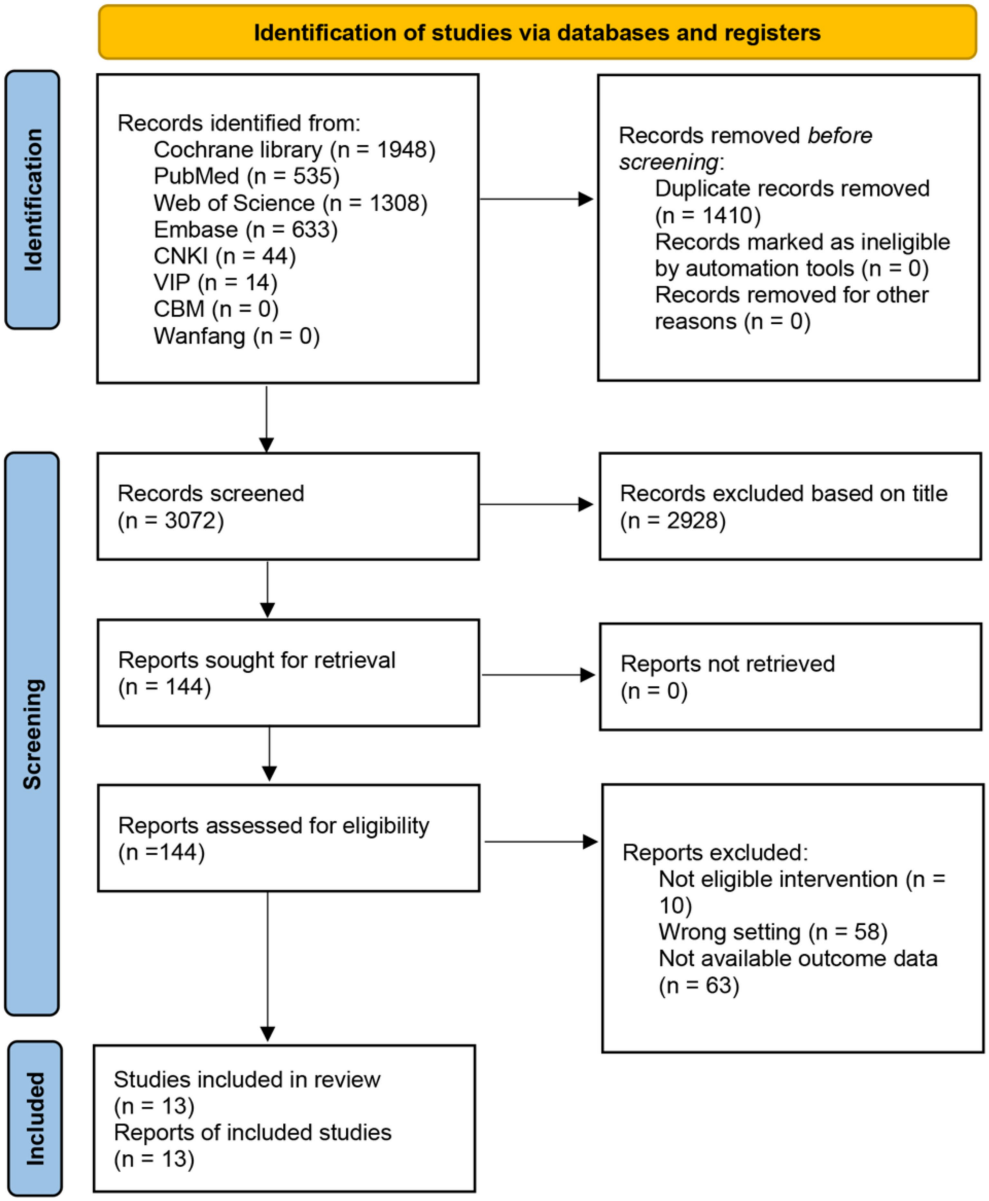
PRISMA flow diagram. PRISMA flow diagram delineates the systematic process of identifying and screening studies across multiple databases, culminating in selecting 13 studies.

Thirteen RCTs (*n* = 612 participants) met all inclusion criteria and provided sufficient data for WMD calculations. The selection process followed PRISMA guidelines (Figure 1), with independent dual-reviewer screening. Study characteristics are summarized in Table 2.

**Table 2:** Characteristics of included studies.

Study	Country	Age		Number of participant (female/male)		Intervention characteristics		Duration	Outcome
		T	C	T	C	T	C		
Amodio et al. (31) (2016)	Italy	55.6 ± 5.0	55.6 ± 5.0	12 (12/0)	11 (11/0)	TRE,8 h + CR	CR	45d	①②③④
Domaszewski et al. (32) (2020)	Poland	65.0 ± 4.0	66 ± 4.7	25 (25/0)	20 (20/0)	TRE,8 h	usual diet	42d	①②⑤⑥
Haganes et al. (33) (2022)	Norway	36.2 ± 5.9	36.4 ± 6.2	21 (21/0)	29 (29/0)	TRE,10 h	usual diet	49d	①③④⑤⑨⑩⑪⑬⑭⑮
Lin et al. (34) (2021)	China	50.1 ± 7.5	54.2 ± 7.9	30 (30/0)	33 (33/0)	TRE,8 h + DCR	CR	56d	①②③④⑥⑧⑨⑩⑪⑫⑬⑭⑮
Domaszewski et al. (35) (2023)	Poland	69.7 ± 3.1	68.8 ± 3.45	29 (29/0)	28 (28/0)	TRE,8 h	usual diet	42d	①②
Irani et al. (36) (2024)	Iran	43.6 ± 9.3	41.0 ± 8.3	29 (29/0)	27 (27/0)	TRE,10 h + CD	CD	56d	①②⑤
Chow et al. (37) (2020)	USA	46.5 ± 12.4	44.2 ± 12.3	11 (9/2)	9 (8/1)	TRE,8 h	usual diet	84d	①③④⑤⑦⑧ ⑨⑩⑫⑬⑭⑮
Cienfuegos et al. (29), TRE4h (2020)	USA	49.0 ± 2.0	45.0 ± 2.0	16 (14/2)	14 (12/2)	TRE,4 h	usual diet	56d	①⑤⑦⑧
Cienfuegos et al. (29), TRE6h (2020)	USA	46.0 ± 3.0	45 ± 2.0	19 (18/1)	14 (12/2)	TRE,6 h	usual diet	56d	①⑤⑦⑧
Thoma et al. (38) (2022)	USA	38.3 ± 7.9	37.8 ± 7.8	41 (34/7)	40 (35/5)	eTRE,10 h + DCR	CR	84d	①⑤⑦
Fagundes et al. (39) (2022)	Brazil	36.2 ± 10.4	31.1 ± 5.6	24 (24/0)	12 (12/0)	TRE,8 h	usual diet	56d	①
Jéssica et al. (30), eTR (2022)	Brazil	33.0 ± 6.0	26.0 ± 4.0	13 (11/2)	13 (11/2)	eTRE, 8 h + CR	CR	56d	①②③④⑤⑥⑨⑩⑪⑫⑬
Jéssica et al. (30), dTR (2022)	Brazil	30.0 ± 7.0	26.0 ± 4.0	11 (9/2)	13 (11/2)	dTRE, 8 h + CR	CR	56d	①②③④⑤⑥⑨⑩⑪⑫⑬
Maruthur et al. (40) (2024)	USA	59.7 ± 7.0	59.1 ± 7.5	21 (19/2)	20 (19/1)	TRE,10 h	usual diet	84d	①②③④⑩⑪⑬⑭⑮
Pureza et al. (41) (2020)	Brazil	31.8 ± 7.0	31.0 ± 0.16	13 (13/0)	14 (14/0)	TRE,12 h + CR	CR	12月	①②⑭⑮

T, Experimental group; C, Control group; M, Male; F, Female; CR, Calorie restriction. ① Weight change. ② BMI. ③ FBG. ④ TG. ⑤ FM. ⑥ FFM. ⑦ LM. ⑧ VFM. ⑨ LDL. ⑩ HDL. ⑪ TC. ⑫ FPI. ⑬ HOMA-IR. ⑭ SBP. ⑮ DBP.

Our systematic review identified 13 randomized controlled trials (RCTs) with a total of 612 participants (intervention: *n* = 315; control: *n* = 297), all providing complete outcome data. Two studies employed three-arm designs: Cienfuegos (29) compared 4-h TRE and 6-h TRE against a shared blank control, and Queiroz (30) compared early TRE and delayed TRE against a shared CR control; these designs yielded 15 pairwise comparisons in total. For synthesis, analyses were stratified by caloric-restriction (CR) status; the dataset comprised seven comparisons of TRE + CR vs. CR and eight comparisons of ad libitum TRE vs. blank control. In multi-arm trials, the shared control was split equally between comparisons to avoid double-counting.

### Confidence in cumulative evidence

**Figure 2:**
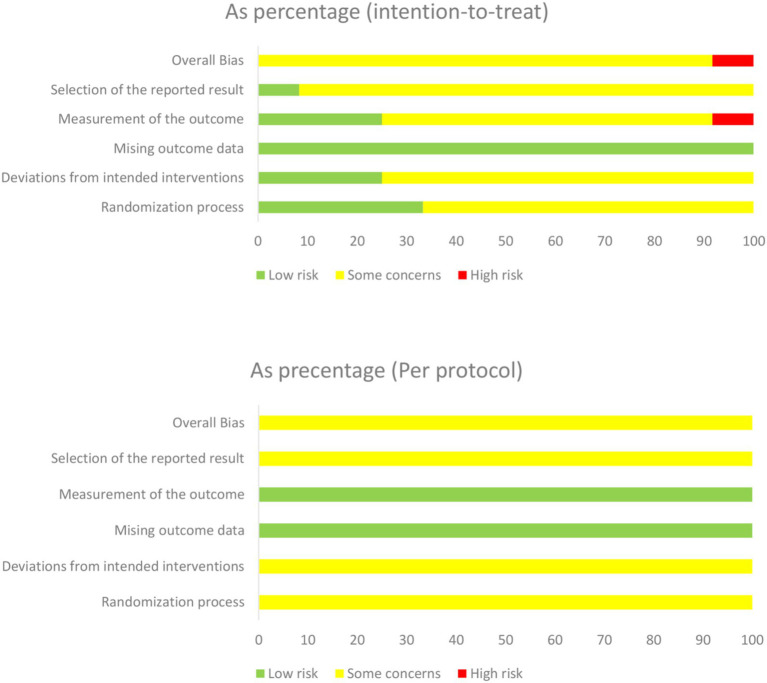
Risk ratio of bias.

**Figure 3:**
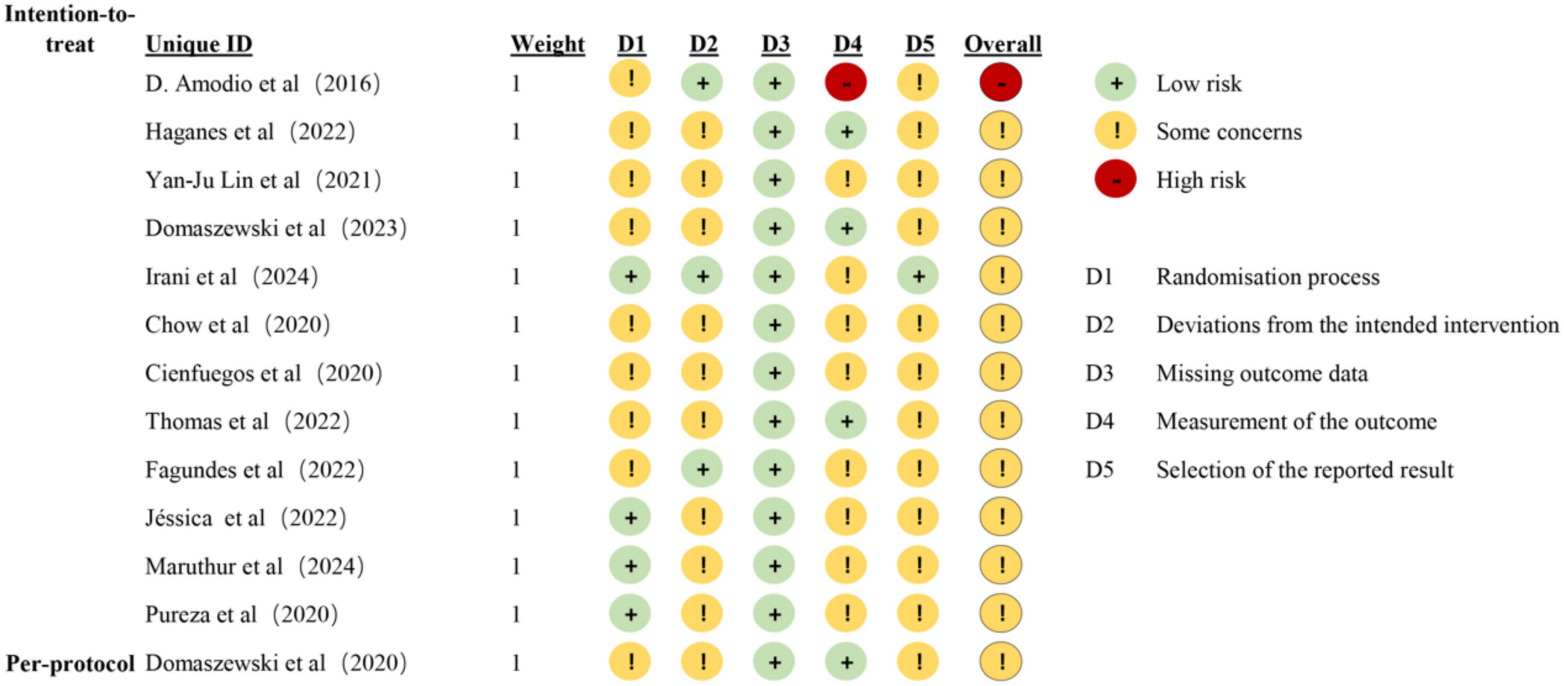
Bias assessment of included literatures.

Results concerning the risk of bias in each domain are shown in Figures 2, 3. Two reviewers (ZXT and CSY) evaluated the RoB using Version 2 of the Cochrane risk-of-bias tool (RoB 2) (25). Most of the studies were considered to carry moderate risk (Supplementary Table S2). One exception was rated as carrying a high risk of bias. The Physiotherapy Evidence Database (PEDro) scale further confirmed study quality, with all included trials scoring ≥ 5 (Supplemental Table S3). For publication bias assessment, funnel plot analysis and Egger’s test showed no significant asymmetry (Supplemental Figure S1).

### Meta-analysis

We performed a systematic meta-analysis examining 15 distinct outcome measures across four physiological domains: body composition, lipid metabolism, glucose metabolism, and blood pressure in the study participants.

#### Effect of time-restricted eating on body composition

##### Effect of time-restricted eating on body weight

The outcomes of 14 randomized controlled trials (29–40) included body weight, with a total sample size of 585. After heterogeneity testing, *I*^2^ = 0.0%, *p* = 0.559, the fixed-effect model was selected for analysis. The results showed that compared with the control group, TRE had a significant effect on weight loss in overweight or obese participants (WMD = −1.927, 95% CI = −3.688 to −0.166, *p* = 0.032), with no publication bias (Egger’s test: *p* = 0.345). In prespecified subgroup analyses by caloric-restriction (CR) status, only body weight showed a differential effect (ad libitum TRE > blank), whereas TRE + CR did not differ from CR and no consistent subgroup effects were observed for other endpoints (Table 3).

**Table 3:** Meta-analysis results of TRE on female body composition and metabolic parameters.

Outcome	Group	Number of study	Number of participant	Heterogeneity		Model	Summary of findings		
				*I* ^2^	*p*	Selection	WMD	95%CI	*p*
Weight	All	14 (29–40)	585	0.0%	0.559	Fixed	−1.927	−3.688, −0.166	0.032*
	TRE	8 (29, 32, 33, 35, 37, 39, 40)	312	12.3%	0.334	Fixed	−2.569	−5.089, −0.048	0.046*
	TRE + HD	6 (30, 31, 34, 36, 38)	273	0.0%	0.676	Fixed	−1.316	−3.776, 1.145	0.295
BMI	All	9 (30–32, 34–36, 40, 41)	362	12.9%	0.327	Fixed	−0.424	−1.068, 0.221	0.197
FBG	All	7 (30, 31, 33, 34, 37, 40)	246	0.0%	0.668	Fixed	0.685	−1.317, 2.687	0.502
TG	All	7 (30, 31, 33, 34, 37, 40)	245	0.0%	0.859	Fixed	2.760	−8.117, 13.637	0.619
FM	All	9 (29, 32, 33, 36–38)	365	0.0%	0.655	Fixed	−1.196	−2.799, 0.407	0.144
FFM	All	4 (30, 32, 34)	158	0.0%	0.881	Fixed	0.417	−1.210, 2.044	0.615
LM	All	4 (29, 37, 38)	164	0.0%	0.662	Fixed	1.212	−1.734, 4.158	0.420
VFM	All	4 (29, 34, 37)	146	0.0%	0.635	Fixed	0.032	−0.209, 0.273	0.795
LDL	All	4 (30, 33, 34, 37)	156	0.0%	0.980	Fixed	−3.214	−12.548, 6.120	0.500
HDL	All	6 (30, 33, 34, 37, 40)	222	21.8%	0.270	Fixed	−1.364	−4.218, 1.489	0.349
TC	All	5 (30, 33, 34, 40)	202	0.0%	0.409	Fixed	4.075	−4.115, 12.264	0.329
FPI	All	5 (30, 33, 34, 37)	181	0.0%	0.504	Fixed	−2.120	−4.172,−0.069	0.043*
HOMA-IR	All	5 (30, 34, 37, 40)	174	0.0%	0.868	Fixed	−0.438	−1.026, 0.149	0.144
SBP	All	5 (33, 34, 37, 40, 41)	201	0.0%	0.469	Fixed	0.377	−3.570, 4.324	0.851
DBP	All	5 (33, 34, 37, 40, 41)	201	36.3%	0.179	Fixed	0.531	−2.482, 3.543	0.730

* indicates statistical significance at *p* < 0.05. Weight, body weight; BMI, body mass index; FBG, fasting blood glucose; TG, triglycerides; FM, fat mass; FFM, fat-free mass; LM, lean mass; VFM, visceral fat mass; LDL, low-density lipoprotein cholesterol; HDL, high-density lipoprotein cholesterol; TC, total cholesterol; FPI, fasting plasma insulin; HOMA-IR, homeostatic model assessment of insulin resistance; SBP, systolic blood pressure; DBP, diastolic blood pressure.

##### Effect of time-restricted eating on body mass index

The outcomes of 9 randomized controlled trials (30–32, 34–36, 40, 41) included BMI, with a total sample size of 362. After heterogeneity testing, *I*^2^ = 12.9%, *p* = 0.327, the fixed-effect model was selected for analysis. The results showed that compared with the control group, TRE had no significant effect on BMI reduction in overweight or obese participants (WMD = −0.424, 95% CI = −1.068 to 0.221, *p* = 0.197), with no publication bias (Egger’s test: *p* = 0.584).

##### Effect of time-restricted eating on fat mass

The outcomes of 9 randomized controlled trials (29, 32, 33, 36–38) included fat mass, with a total sample size of 365. After heterogeneity testing, *I*^2^ = 0.0%, *p* = 0.655, the fixed-effect model was selected for analysis. The results showed that compared with the control group, TRE had no significant effect on fat mass reduction in overweight or obese participants (WMD = −1.196, 95% CI = −2.799 to 0.407, *p* = 0.144), with no publication bias (Egger’s test: *p* = 0.150).

##### Effect of time-restricted eating on fat-free mass

The outcomes of 4 randomized controlled trials (30, 32, 34) included fat-free mass, with a total sample size of 158. After heterogeneity testing, *I*^2^ = 0.0%, *p* = 0.881, the fixed-effect model was selected for analysis. The results showed that compared with the control group, TRE had no significant effect on fat-free mass in overweight or obese participants (WMD = 0.417, 95% CI = −1.210 to 2.044, *p* = 0.615), with no publication bias (Egger’s test: *p* = 0.092).

##### Effect of time-restricted eating on lean body mass

The outcomes of 4 randomized controlled trials (29, 37, 38) included lean body mass, with a total sample size of 164. After heterogeneity testing, *I*^2^ = 0.0%, *p* = 0.662, the fixed-effect model was selected for analysis. The results showed that compared with the control group, TRE had no significant effect on lean body mass in overweight or obese participants (WMD = 1.212, 95% CI = −1.734 to 4.158, *p* = 0.420), with no publication bias (Egger’s test: *p* = 0.930).

##### Effect of time-restricted eating on visceral fat mass

The outcomes of 4 randomized controlled trials (29, 34, 37) included visceral fat mass, with a total sample size of 146. After heterogeneity testing, I^2^ = 0.0%, *p* = 0.635, the fixed-effect model was selected for analysis. The results showed that compared with the control group, TRE had no significant effect on visceral fat mass in overweight or obese participants (WMD = 0.032, 95% CI = −0.209 to 0.273, *p* = 0.795), with no publication bias (Egger’s test: *p* = 0.467).

#### Effect of time-restricted eating on lipid metabolism

##### Effect of time-restricted eating on triglycerides

The outcomes of 7 randomized controlled trials (30, 31, 33, 34, 37, 40) included triglycerides, with a total sample size of 245. After heterogeneity testing, *I*^2^ = 0.0%, *p* = 0.859, the fixed-effect model was selected for analysis. The results showed that compared with the control group, TRE had no significant effect on triglyceride levels in overweight or obese participants (WMD = 2.760, 95% CI = −8.117 to 13.637, *p* = 0.619), with no publication bias (Egger’s test: *p* = 0.860).

##### Effect of time-restricted eating on low-density lipoprotein

The outcomes of 4 randomized controlled trials (30, 33, 34, 37) included low-density lipoprotein levels, with a total sample size of 156. After heterogeneity testing, *I*^2^ = 0.0%, *p* = 0.980, the fixed-effect model was selected for analysis. The results demonstrated that compared with the control group, TRE showed no significant effect on LDL cholesterol levels in overweight or obese participants (WMD = −3.214, 95% CI = −12.548 to 6.120, *p* = 0.500), with no evidence of publication bias (Egger’s test: *p* = 0.124).

##### Effect of time-restricted eating on high-density lipoprotein

The outcomes of 6 randomized controlled trials (30, 33, 34, 37, 40) included high-density lipoprotein, with a total sample size of 222. After heterogeneity testing, *I*^2^ = 21.8%, *p* = 0.270, the fixed-effect model was selected for analysis. The results showed that compared with the control group, TRE had no significant effect on high-density lipoprotein levels in overweight or obese participants (WMD = −1.364, 95% CI = −4.218 to 1.489, *p* = 0.349), with no publication bias (Egger’s test: *p* = 0.089).

##### Effect of time-restricted eating on total cholesterol

The outcomes of 5 randomized controlled trials (30, 33, 34, 40) included total cholesterol, with a total sample size of 202. After heterogeneity testing, *I*^2^ = 0.0%, *p* = 0.409, the fixed-effect model was selected for analysis. The results showed that compared with the control group, TRE had no significant effect on total cholesterol levels in overweight or obese participants (WMD = 4.075, 95% CI = −4.115 to 12.264, *p* = 0.329), with no publication bias (Egger’s test: *p* = 0.478).

#### Effect of time-restricted eating on glucose metabolism

##### Effect of time-restricted eating on fasting blood glucose

The outcomes of 7 randomized controlled trials (30, 31, 33, 34, 37, 40) included fasting blood glucose, with a total sample size of 246. After heterogeneity testing, *I*^2^ = 0.0%, *p* = 0.668, the fixed-effect model was selected for analysis. The results showed that compared with the control group, TRE had no significant effect on fasting blood glucose levels in overweight or obese participants (WMD = 0.685, 95% CI = −1.317 to 2.687, *p* = 0.502), with no publication bias (Egger’s test: *p* = 0.123).

##### Effect of time-restricted eating on fasting plasma insulin

The outcomes of 5 randomized controlled trials (30, 33, 34, 37) included fasting plasma insulin, with a total sample size of 181. After heterogeneity testing, *I*^2^ = 0.0%, *p* = 0.504, the fixed-effect model was selected for analysis. The results demonstrated that compared with the control group, TRE significantly reduced fasting plasma insulin levels in overweight or obese participants (WMD = −2.120, 95% CI = −4.172 to −0.069, *p* = 0.043), with no publication bias (Egger’s test: *p* = 0.485).

##### Effect of time-restricted eating on homeostasis model assessment of insulin resistance

The outcomes of 5 randomized controlled trials (30, 34, 37, 40) included insulin resistance, with a total sample size of 174. After heterogeneity testing, *I*^2^ = 0.0%, *p* = 0.868, the fixed-effect model was selected for analysis. The results showed that compared with the control group, TRE had no significant effect on HOMA-IR in overweight or obese participants (WMD = −0.438, 95% CI = −1.026 to 0.149, *p* = 0.144), with no publication bias (Egger’s test: *p* = 0.777).

#### Effect of TRE on blood pressure

##### Effect of time-restricted eating on systolic blood pressure

The outcomes of 5 randomized controlled trials (33, 34, 37, 40, 41) included systolic blood pressure, with a total sample size of 201. After heterogeneity testing, *I*^2^ = 0.0%, *p* = 0.469, the fixed-effect model was selected for analysis. The results showed that compared with the control group, TRE had no significant effect on systolic blood pressure in overweight or obese participants (WMD = 0.377, 95% CI = −3.570 to 4.324, *p* = 0.851), with no publication bias (Egger’s test: *p* = 0.389). Effect of time-restricted eating on diastolic blood pressure (DBP).

The outcomes of 5 randomized controlled trials (33, 34, 37, 40, 41) included diastolic blood pressure, with a total sample size of 201. After heterogeneity testing, *I*^2^ = 36.3%, *p* = 0.179, the fixed-effect model was selected for analysis. The results demonstrated that compared with the control group, TRE had no significant effect on diastolic blood pressure levels in overweight or obese participants (WMD = 0.531, 95% CI = −2.482 to 3.543, *p* = 0.730), with no evidence of publication bias (Egger’s test: *p* = 0.081).

## Discussion

### TRE contributes to weight management in overweight and obese women

Obesity elevates mortality risks associated with various diseases and increases the likelihood of developing multiple comorbidities. Our findings demonstrate that TRE shows superior efficacy in weight regulation compared to a regular diet among overweight and obese women, the pooled effect aligns with recent meta-analyses showing modest losses, likely reflecting spontaneous reductions in energy intake rather than meal timing under isocaloric conditions (42). Animal studies by Chaix et al. (43, 44) have demonstrated that TRE prevents and reverses adverse metabolic outcomes induced by various nutritional challenges, including obesogenic diets high in fat and sucrose. Compared to high-fat diet-fed mice, TRE-treated mice demonstrated significant reductions in obesity indices and hepatic steatosis, along with improved glucose tolerance and lowered cholesterol levels. These metabolic alterations may indicate enhanced homeostatic regulation across multiple tissue systems. In the general population, daily eating patterns typically span 15 h or longer. The desynchronization between circadian biology and behavioral patterns, particularly food consumption misaligned with endogenous circadian phases, has been identified as a key determinant of obesity and metabolic disorder risks (45). TRE facilitates circadian rhythm realignment through regulated eating-fasting cycles and avoidance of nocturnal caloric intake (46). Furthermore, human participants under TRE protocols have been observed to voluntarily reduce their caloric intake (47).

A key aspect of this study is the subgroup analysis of TRE variants based on CR status. TRE protocols are generally classified as “ad libitum” or “prescribed” (48). “Ad libitum” TRE allows unrestricted food consumption without caloric restriction (CR) during specified feeding windows. In contrast, prescribed TRE entails adherence to specific guidelines regarding food selection or caloric intake during feeding windows. Subgroup analysis demonstrated that TRE alone showed significant weight-loss effects compared to blank controls, whereas the combination of TRE with CR showed no statistically significant difference compared to CR alone. This suggests that TRE’s metabolic effects may not surpass those of daily CR in obese individuals (49), with CR accounting for the majority of benefits observed in time-restricted feeding protocols. This interpretation is supported by findings from isocaloric trials, which report no significant difference in weight loss between TRE and traditional calorie-restricted diets when total energy intake is matched (50). These findings highlight the importance of considering distinct characteristics across TRE methodologies. Future research should prioritize standardization of controlled dietary interventions to enable direct comparisons and establish more conclusive findings (48).

### Time-restricted eating can effectively lower insulin levels in overweight and obese women

TRE demonstrates efficacy in reducing insulin levels among women with overweight or obesity. As a primary metabolic hormone secreted by pancreatic *β*-cells, insulin primarily regulates glucose homeostasis by promoting cellular glucose uptake and glycogen synthesis. Insulin resistance, characterized by diminished sensitivity to insulin’s metabolic actions, is mediated by genetic predisposition and environmental determinants. This metabolic dysfunction constitutes a principal risk factor for type 2 diabetes mellitus, hypertension, dyslipidemia, and atherosclerotic cardiovascular pathologies (51). In our meta-analysis, TRE lowered fasting insulin and HOMA-IR, in line with prior reports (52–54). Mechanistically, constraining the eating window reduces the number and duration of insulinogenic exposures and limits late-evening intake, thereby avoiding the melatonin–dinner mismatch and blunted *β*-cell responsiveness (55, 56). Shorter eating hours also reduce snacking opportunities; in practice, ad libitum TRE often produces modest spontaneous energy-intake reductions, further decreasing insulin demand (42). Over weeks, prolonged nightly fasting increases fatty-acid oxidation/ketogenesis and may enhance hepatic insulin signaling and insulin clearance, changes consistent with the observed reductions in fasting insulin (57, 58). Previous investigations into prolonged fasting protocols have documented weight reduction, enhanced insulin sensitivity, improved sleep parameters, and attenuated inflammatory responses (37, 59–62). This interpretation is consistent with recent syntheses attributing early metabolic gains to longer fasting-induced energy deficits and circadian alignment rather than timing alone under isocaloric prescriptions (42).

### Time-restricted eating demonstrates favorable safety and compliance profiles

While weight loss remains the primary intervention target for overweight and obese individuals, concomitant body composition changes merit equal attention. Existing evidence indicates that daily CR-induced weight loss comprises approximately 75–80% fat mass reduction and 20–25% FFM loss (63). As a key determinant of basal metabolic rate, FFM plays crucial roles in metabolic regulation, skeletal integrity maintenance, and functional capacity preservation (64). FFM reduction may accelerate age-related strength decline in older women. Moreover, CR-induced weight loss may reduce both resting metabolic rate (RMR) and sympathetic nervous system (SNS) activity, potentially predisposing women to weight regain (65). Our findings reveal that weight loss observed during TRE interventions did not induce additional reductions in fat-free mass or lean body mass among female participants. This finding aligns with previous observations in healthy male cohorts. In this protocol, participants maintained daily 16-h fasting windows while continuing regular resistance training. The intervention resulted in preserved lean body mass and maximal strength alongside body fat reduction in male TRE participants (66). Within the included studies, only one participant discontinued due to inability to adhere to the prescribed feeding window (37). Four trials (29, 30, 33, 34) documented mild transient adverse events, including dizziness, headaches, nausea, and diarrhea. These symptoms typically occurred during the initial intervention phase and resolved spontaneously over time. Collectively, these findings suggest superior compliance and safety profiles of TRE compared to CR interventions.

### Metabolic effects of time-restricted eating in overweight and obese women

Our findings indicate that TRE significantly improves insulin levels in overweight and obese women, whereas no significant alterations were observed in blood pressure, glucose metabolism, or lipid profiles. Consistent with these observations, clinical evidence for lipid outcomes in women remains inconclusive, with contradictory findings across trials (67). Notably, experimental evidence demonstrating metabolic benefits predominantly originates from male rodent models (68, 69) or small-scale human clinical trials (70). Taken together, these observations suggest that not all TRE schedules are physiologically equivalent (71, 72). Population data also indicate a non-linear association between nocturnal fasting duration and cardiometabolic indicators: both shorter (<10.0 h) and longer (>14.1 h) fasting durations were independently associated with higher age-related markers in NHANES (73). This schedule-dependence underscores the need to delineate the timing, duration, and stability of the eating window when evaluating metabolic endpoints (55, 74).

These findings imply that the health impacts of TRE’s fasting window may involve complex mechanisms requiring further investigation. Future research should prioritize large-scale clinical studies with rigorous designs to objectively determine TRE’s effects on glucose metabolism, lipid profiles, and blood pressure regulation.

### Strengths and limitations

This systematic review specifically focused on TRE as a distinct dietary intervention and restricted inclusion to overweight and obese female populations, thereby enhancing the clinical relevance and sex-specific interpretability of the findings. By separately analyzing anthropometric and metabolic parameters, and employing standardized tools for quality assessment and evidence grading, the study provides a methodologically robust synthesis of TRE’s effects in this demographic. Nonetheless, several limitations should be acknowledged that may influence the generalizability and interpretation of the findings. First, the relatively small number of eligible trials—together with our eligibility criterion restricting inclusion to women-only cohorts or mixed-sex cohorts with ≥85% female participants to preserve analytic focus—resulted in a limited total sample size; accordingly, the findings are primarily applicable to women. Second, the inherent design characteristics of the included studies precluded implementation of blinding procedures. Third, substantial heterogeneity in outcome measurements across primary studies and the limited number of randomized controlled trials reporting secondary outcomes precluded quantitative assessment of publication bias through funnel plot analyses for most endpoints. Collectively, these limitations underscore the necessity for methodologically robust studies with larger sample sizes and extended follow-up periods to comprehensively evaluate TRE interventions in female populations. We also acknowledge that heterogeneity in TRE protocols, such as differences in feeding window duration, timing, and caloric restriction, limited our ability to perform subgroup analyses. We emphasize the need for future trials to directly compare different TRE regimens in women.

Most outcomes had low heterogeneity (I^2^ < 25%). Where ≥10 trials were available, funnel plots were roughly symmetric and Egger’s tests were non-significant (Supplementary Figure S1), though limited study numbers mean small-study/publication bias cannot be excluded. We encourage precise TRE reporting—clock-defined daily window (start/end, length), caloric strategy, adherence with monitoring, co-interventions/comparators, and standardized outcomes—to improve inclusion and comparability across trials.

## Conclusion

The principal findings are as follows: (1) Time-restricted eating may serve as an effective intervention for overweight and obese women, demonstrating weight reduction and lower fasting insulin while preserving both fat-free mass and lean body mass. (2) When compared to traditional calorie-restricted diets, time-restricted eating shows no statistically significant differences in weight loss outcomes. (3) Compared to conventional low-calorie diets, time-restricted eating exhibits a more favorable safety profile and higher adherence rates. (4) The long-term metabolic impacts of time-restricted eating in overweight and obese female populations warrant further investigation.

Clinically, in overweight and obese women, time-restricted eating can be offered as an adherence-friendly alternative that achieves modest weight loss without compromising lean mass; however, definitive guidance will require isocaloric, protocol-standardized trials with long-term follow-up.

## Data Availability

The original contributions presented in the study are included in the article/Supplementary material, further inquiries can be directed to the corresponding author.
